# Early COVID-19 infection after lung transplantation in a patient with cystic fibrosis

**DOI:** 10.6061/clinics/2020/e2274

**Published:** 2020-11-25

**Authors:** Rodrigo Abensur Athanazio, André Nathan Costa, Rafael Medeiros Carraro, Diego Gonzalez, Samia Zahi Rached, Marcos Naoyuki Samano, Ricardo Henrique de Oliveira Braga Teixeira, Silvia Vidal Campos

**Affiliations:** I Divisao Pulmonar, Instituto do Coracao (InCor), Hospital das Clinicas (HCFMUSP), Faculdade de Medicina, Universidade de Sao Paulo, Sao Paulo, SP, BR; II Divisao de Cirurgia Toracica, Instituto do Coracao (InCor), Hospital das Clinicas (HCFMUSP), Faculdade de Medicina, Universidade de Sao Paulo, Sao Paulo, SP, BR

Since December 2019, when the initial cases of severe viral pneumonia caused by a new coronavirus were described, health services worldwide have sought to understand the evolution and risk factors related to the severity of coronavirus disease (COVID-19). Elderly individuals have been shown to be the highest risk group. However, other factors, such as male sex; obesity; smoking; and chronic heart, lung, kidney, and liver diseases, are also associated with a poor prognosis. We draw attention to immunosuppressed patients as they are a vulnerable group with a high susceptibility to severe forms of the disease (1,2). Clinical data on COVID-19 infection in a lung transplant population are still very limited. Herein, we report on a severe presentation of severe acute respiratory syndrome coronavirus 2 (SARS-CoV-2) infection in a 37-year-old male patient with cystic fibrosis (CF) in the second week after lung transplantation (LTx).

The patient was diagnosed as having CF at 7 months of age (homozygous F508del). Chronic airway infections included those caused by methicillin resistant *Staphylococcus aureus*, carbepenem-resistent *Pseudomonas aeruginosa*, and *Aspergillus* sp.. Because of his progressive respiratory insufficiency, bilateral LTx was performed in April 2020. The surgery was performed with 315 minutes of ischemic time, and there was no need for cardiopulmonary bypass. The donor was a 21-year-old man without comorbidities, without pulmonary infiltrates on chest computed tomography (CT), and with a negative SARS-CoV-2 nasopharyngeal swab polymerase chain reaction (PCR) who had developed encephalic death, confirmed 72 hours after a traumatic brain injury.

The chosen antimicrobial prophylaxis was piperacillin-tazobactam plus colistin, teicoplanin, voriconazole, cotrimoxazole, and ganciclovir. Induction immunosuppression was performed with basiliximab followed by cyclosporine, sodium mycophenolate, and prednisone as maintenance therapy. Transbronchial biopsy on post-operative day (POD) 9 revealed acute rejection (A2Bx) (A2: mild acute rejection characterized by perivascular and interstitial mononuclear infiltrates; Bx: ungradable small airway inflammation), and a 3-day course of methylprednisolone 10 mg/kg/d was started on POD 11. The patient presented with hypoxemia on the first day of acute cellular rejection treatment with methylprednisolone. Pulmonary thromboembolism (PTE) was considered, and a chest CT scan was performed. Nasopharyngeal swabs for COVID-19 were collected after ground-glass opacities were observed on chest CT with no signs of PTE. Chest CT also showed areas of consolidation in the lower right lobe and bilateral pleural effusion (Figure 1A). The patient rapidly progressed to severe hypoxemia, despite having only mild dyspnea and no fever. Because of respiratory distress, mechanical ventilation was required 3 days after the start of methylprednisolone pulse therapy. According to the standard of care at our institute, protective ventilatory support was initiated, with low tidal volume (6 ml/kg) and driving pressure below 15 cmH_2_0. Prone positioning was not required. The tracheal aspirate sample was positive for both adenovirus and SARS-CoV-2. Immunosuppression was managed by reducing methylprednisolone to 0.5 mg/kg, suspending mycophenolate, and switching from cyclosporin to tacrolimus. He had a good clinical outcome without the use of antivirals or immunomodulatory drugs. He was weaned off invasive mechanical ventilation on POD 19 and was discharged from the intensive care unit on POD 23. He presented with progressive clinical and radiological improvement (Figure 1B) in the ward and was discharged from hospital on POD 39, although he still tested positive on SARS-CoV-2 PCR tests at 15 and 25 days after symptom onset.

Immunosuppressed patients might be a vulnerable group and are more susceptible to severe forms of the disease, as in past epidemics such as H1N1 and SARS-CoV-1, wherein a higher mortality rate because of viral respiratory infections was documented. To date, few cases of COVID-19 in LTx patients have been reported, of which 13 were in CF recipients, with a mean age of 40 years, and occurred 7 years after LTx. All had good clinical outcomes and recovered completely (1,2,4).

In this report, we highlight a case of COVID-19 after two weeks of LTx in a LTx recipient undergoing methylprednisolone pulse therapy due to acute cellular rejection. Particularly rapid evolution of the disease was noted in the patient, who required mechanical ventilation 72 hours after the onset of symptoms. Previous reports with non-transplant patients have shown that COVID-19 usually presents with more severe symptoms and respiratory insufficiency between 10 and 14 days after symptom onset (3).

Our patient also had an adenovirus infection detected on PCR. Co-infection with SARS-CoV-2 and other respiratory viruses has already been reported, and it can occur in as high as in 20% of all cases tested (5). A Spanish group reported four COVID-19 cases co-infected with influenza (6), and one French LTx patient had co-infection with rhinovirus (3). Despite viral co-infection, these cases had a similar evolution to that of other COVID-19 cases. Nevertheless, adenovirus infection may be associated with severe respiratory symptoms in LTx patients. We hypothesized that viral co-infection and the short time after LTx could explain his rapid clinical deterioration, marked by precocious spreading of the virus to lung allografts within a scenario of intense immunosuppression concomitant with inflammatory tissue damage due to rejection.

Our patient showed clinical improvement with supportive treatment without any directed treatment for COVID-19. Although the most probable source of infection was nosocomial, we were not able to determine whether it was from a healthcare professional or the patient’s caregivers, as access to the patient was not restricted when the patient’s condition was worsening. Nosocomial infection, especially in countries with a high rate of SARS-CoV-2 infection, must be prevented. It is very unlikely that the donor was the source of the infection. In addition to the donor having tested negative for SARS-CoV-2 before LTx, an incubation period of two weeks further disfavored this hypothesis in our immunosuppressed patient. In this case, nasopharyngeal swabs of the recipient were not collected before the LTx. Currently, per institute protocols, nasopharyngeal swabs are collected for SARS-CoV-2 PCR testing from all patients. Further, we decided to prioritize cases (severe respiratory failure) for management during the acceleration phase of the pandemic in Brazil and attempted to perform chest CT of the donor before organ harvesting. Although the risk of SARS-CoV-2 infection is high and potentially severe, these patients are also at risk for pulmonary exacerbation and death from their underlying disease. Pulmonary transplantation activities should be maintained with special attention to donor surveillance and post-operative management. Further studies are needed to better understand the role of co-infections with other viruses and the impact of immunosuppression intensity on the clinical outcomes of LTx recipients with COVID-19. This new respiratory viral infection also needs to be considered as a potential risk factor for chronic lung allograft dysfunction.

## Figures and Tables

**Figure 1:**
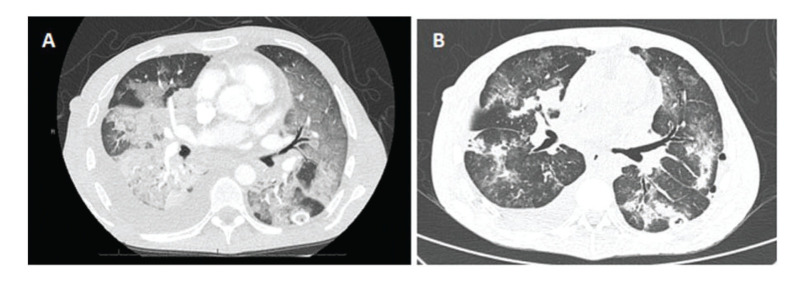
**A)** Chest computed tomography (CT) on post-operative day (POD) 12, one day before COVID-19 diagnosis, marked by bilateral alveolar opacities characterized as consolidation and ground-glass opacity areas covering more than 75% of the lungs **B)** Chest CT on POD 25 showing bilateral consolidation and ground-glass opacity areas under high resolution.
